# Detection of EWS/FLI-1 fusion in non-Ewing soft tissue tumors

**Published:** 2015

**Authors:** IO Trancău, R Huică, M Surcel, A Munteanu, C Ursaciuc

**Affiliations:** * "Foişor” Orthopedics Clinical Hospital, Bucharest, Romania; ** Immunology Department, “Victor Babeş” National Institute of Pathology, Bucharest, Romania; *** “Carol Davila” University of Medicine and Pharmacy, Bucharest, Romania

**Keywords:** EWS/FLI-1 fusion, soft tissue tumors, genomic investigation

## Abstract

**Objectives:** EWS/FLI-1 fusion mainly appears in Ewing’s sarcoma or the primitive neuroectodermal tumors and represents a genomic marker for these tumors. However, it can appear with lower frequency in other soft tissue tumors. The paper investigates the presence of EWS/FLI-1 fusion in clinically diagnosed sarcoma belonging to different non-Ewing connective tissue tumors in order to search for a possible new biomarker valuable for investigators.

**Methodology:** 20 patients with soft tissue tumors, who underwent surgery, were tested. Intra-operative samples of normal and tumor tissue were collected for histopathological diagnosis and genetics determinations. The patients’ RNA from tumor and normal peritumoral tissue was extracted and EWS/FLI-1 fusion screened by quantitative real-time PCR. The relative expression of the fusion in the tumor sample was compared to the similar expression in normal tissue.

**Results:** The amplification in the threshold zone was shown by 5 samples (25%): 2 clear cell sarcoma, 1 fibrosarcoma, 1 malignant tumor of nerve sheath, 1 metastatic adenocarcinoma. We differentiated between the unspecific amplification and concluded that these are weak positive results.

**Conclusions:** Genomic investigation may establish the tumor malignancy and its possible affiliation earlier than histopathology. It can support the screening of EWS/FLI-1 fusion in a larger variety of clinically diagnosed soft tissue tumors.

## Introduction

The chromosomal translocation t(11;22)(q24,q12) or EWS/FLI-1 fusion is mainly developed in Ewing’s sarcoma (ES) or primitive neuroectodermal tumors (PNET) and can represent a genomic diagnostic marker for these tumors [**1**-**3**]. The translocation results in the fusion of the N-terminal region of the EWS (Ewing’s sarcoma) gene with the DNA binding domain of the proto-oncogene FLI-1 (Friend leukemia integration site 1) [**4**]. The EWS gene is located on chromosome 22, encoding a protein that couples RNA, while FLI-1 gene is localized on chromosome 11, being a member of the ETS (E twenty-six) family of transcription factors. EWS protein in normal cells is involved in gene expression, after binding to RNA [**5**], and FLI-1 protein is attached to DNA and regulates transcription, controlling cell growth [**6**]. The translocation t(11, 22) is followed by the formation of a chimeric protein, EWS1/FLI-1, which influences the transcription, generating uncontrolled cell division and abnormal maturation and leads to malignant transformation [**7**-**9**]. This fusion protein has the potential to promote tumorigenesis by activating the transcription of an aberrant FLI-1 factor [**10**,**11**].

In terms of EWS/FLI-1 translocation, positive cells present a gene amplification detectable by RT-PCR in the bone marrow and peripheral blood of patients with ES and PNET with or without metastasis [**12**]. The molecular detection of EWS/FLI-1 fusion is valuable in the differential diagnosis of small cell tumors and ES staging and prognosis [**13**]. Although it is characteristic of ES, the fusion can appear with a lower frequency in soft-tissue sarcoma with clear cells (soft tissue melanoma) [**14**,**15**] or Ewing family tumors (neuroectodermal tumors, bone sarcomas) [**16**].

Considering that clinical features of ES may resemble those of the other types of sarcomas or some adenocarcinoma tumors, it is interesting to study the presence of the fusion in clinically diagnosed sarcomas which belong to different types of connective tissue tumors. In this study, we tried to detect EWS/FLI-1 fusion in non-Ewing soft tissue tumors, in order to search for a possible new biomarker valuable for investigators.

## Materials and Methods

**Patients**

20 patients with soft tissue tumors, hospitalized in “Foişor” Orthopedics Clinical Hospital in Bucharest were tested during January 2013-May 2014. All the patients underwent surgery, and intra-operative samples of tumor tissue and normal peritumoral tissue were collected for histopathological diagnosis and genetics determinations.

**Table 1** presents the distribution of cases in the study in terms of final histopathological diagnosis.

**Table 1:** Investigated casuistry

Type of tumor *	**No. of cases**
Liposarcoma	2
Giant cell tumor	2
Fibrosarcoma	2
Malignant tumors of nerve sheath	2
Clear cell sarcoma	2
Undifferentiated sarcoma	1
Metastatic adenocarcinoma	3
Multiple myeloma	1
Benign tumors	5
* Histopathological diagnosis	

All the subjects gave an informed consent before their inclusion in the study. The study was approved by the institution’s ethics committee.

**RNA extraction from biological samples**

Fresh harvested tumor and normal tissue were immediately placed in 1 ml cryovials (Nalgene Nunc, Penfield, NY, USA) with RNA later stabilizer (Qiagen GmbH, Hilden, Germany) and maintained overnight at 2-8°C. After removing the stabilizing reagent, tissues were kept at -80°C (ScienTemp freezer, Adrian, MI, USA).

The RNA extraction was performed with RNeasy Mini Kit (Qiagen Sciences, Maryland, USA). Approximately 30 mg of tissue were suspended in 600µL lysis buffer (Buffer RLT) and homogenized with a TissueRuptor homogenizer. The lysate was subsequently centrifuged for 3 minutes at 10,000 g and the supernatant was transferred by pipetting into a microcentrifuge tube, with 600µL 70% ethanol (Chimopar, Bucharest, Romania). The sample was then transferred to a separation column (RNeasy spin column) and centrifuged at 8000 g in RW1 Buffer (15 sec.) and RPE Buffer (15 sec. and 2 min.). RNA elution was performed with 50µL nuclease-free water by 1 min. centrifugation at 8000 g.

The removal of the contaminating genomic DNA was performed with 2U DNase Turbo for 10µg RNA in 50µL volume and was followed by a treatment with DNase inactivation agent (Turbo DNA free kit, Ambion - Life Technologies, Carlsbad, CA, USA).

The RNA extracts concentration was determined by UV absorbance at 230-350 nm in EPOCH Multi-Volume spectrophotometer (Biotek, Winooski, VT, USA). The extract quality was determined based on the OD260/OD280 optical densities ratio (with an average value of 1.94 ± 0.23). After the determinations, all the RNA samples extracted were considered adequate in terms of quality and quantity.

**Detection of EWS/FLI-1 fusion**

EWS/FLI-1 fusion was screened by quantitative real-time PCR (qPCR) with Rotor Gene 6000 thermocycler (Corbett Life Science, Qiagen Sciences, Maryland, USA) using the TaqMan RNA-to-Ct 1 Step Kit (Applied Biosystems - Life Technologies, Carlsbad, CA, USA), with Hs03024497_ft primer specific for fusion. PCR data normalization was performed by using 18S rRNA gene reference and Hs03928990_g1 primer. A RNA sample from the tumor tissue and a RNA sample from the normal peritumoral tissue were analyzed in duplicate in each case. Each set of determinations included a duplicate with no complementary DNA negative control (NTC). The thermal cycling conditions (profile of polymerization reaction) are shown in **Table 2**.

**Table 2:** Thermal cycling conditions (profile of polymerization reaction)

**Stage**	**Step**	**Temp (°C)**	**Time**	**No. of cycles **
Holding	Reverse-transcription	48	15 min.	1
Holding	Activation of AmpliTaq Gold® DNA Polymerase	95	10 min.	1
Cycling	Denature	95	15 sec.	40
	Anneal/Extend	60	1 min. Fluorescent signaling detection	

Data analysis was performed with Rotor-Gene 6000 software (Corbett Life Science), determining the relative expression of EWS1/FLI-1 fusion in the test sample (with possibly fusion) compared to the similar expression in normal tissue (ΔΔCt method).

## Results

**Fig. 1:**
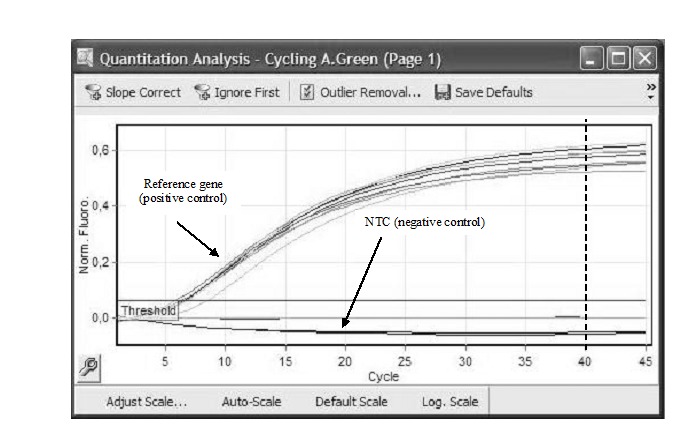
Quantitative analysis on Green fluorescence channel for reference genes and NTC RT-qPCR at RotorGene 6000 termocycler with TaqMan RNA-to-Ct 1 Step Kit and Hs03928990_g1- TaqMan Gene Expression Assay. The cycling kit threshold (Ct) is featured pointed

The fluorescence signals obtained for NTC, internal controls (reference genes), normal tissue RNA sample and tumor tissue RNA sample were analyzed in Green fluorescence channel. All reference genes were amplified with signals in Green fluorescence channel, while the NTC negative controls did not have a positive fluorescent signal (**Fig. 1**). Thus, the experiments were considered valid.

**Fig. 2:**
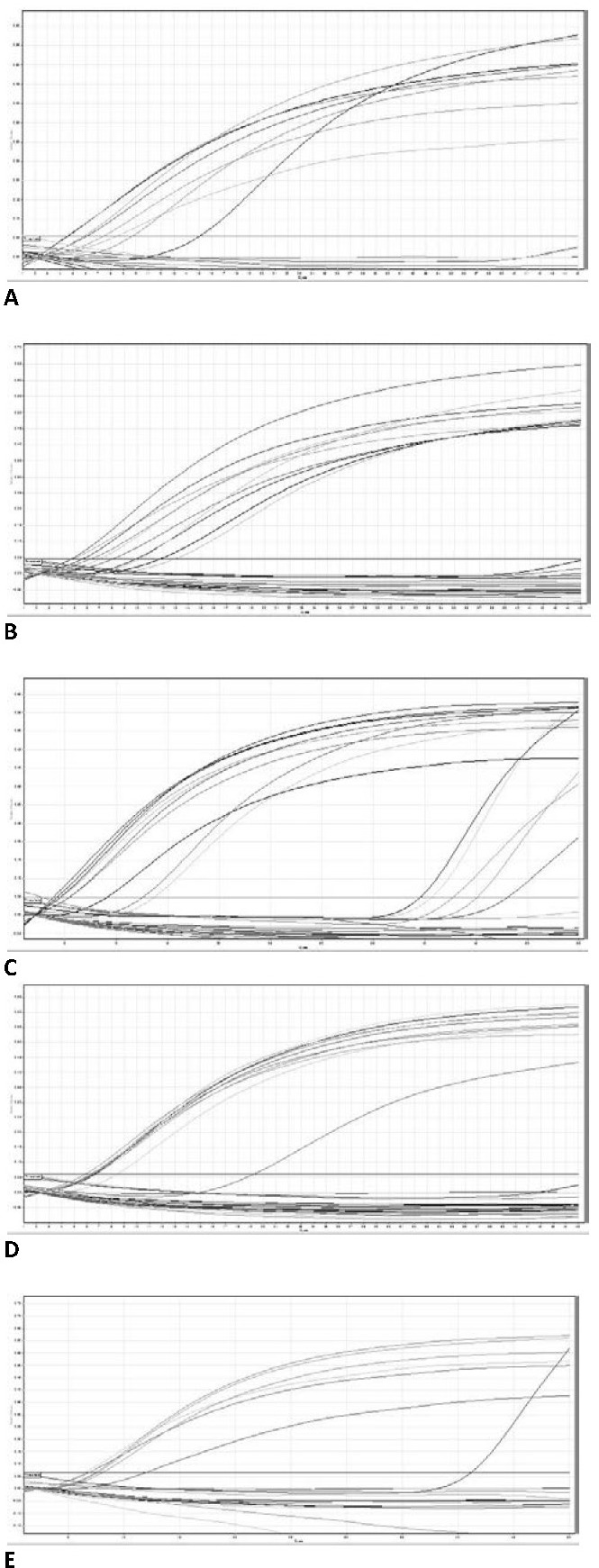
Quantitative analysis on Green fluorescent channel for RNA samples (normal tissue RNA / tumor tissue RNA). a. Cases no. 3, 4; b. Cases no. 5-9; c. Cases no. 10-14; d. Cases no. 15-19; e. Cases no. 20-22 RT-qPCR at RotorGene 6000 termocycler with TaqMan RNA-to-Ct 1 Step Kit and Hs03024497_ft - TaqMan Gene Expression Assay

**Fig. 3:**
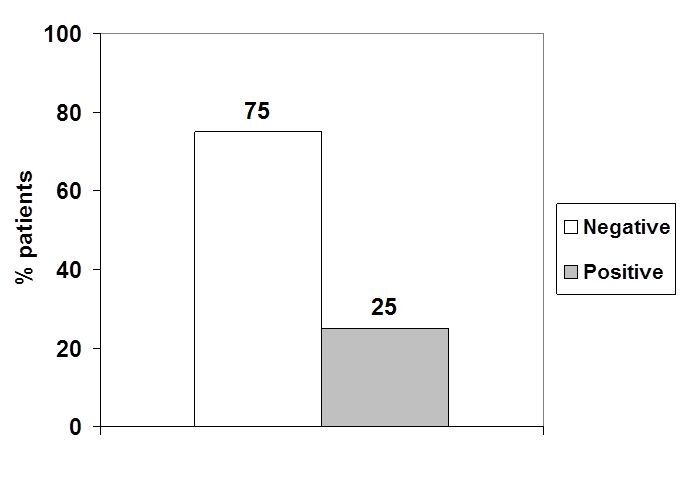
Distribution of EWS/FLI-1 fusion in studied cases RT-qPCR practiced on RNA samples from 20 patients with soft tissue tumors

The patients RNAs were analyzed by qPCR for EWS/FLI-1 fusion (**Fig. 2**). Fluorescent signals in the threshold zone were shown by 5 samples (25%) (**Fig. 2c,2e**,**Fig. 3**). In these patients, the Cycle threshold (Ct) values were located around the kit Ct (**Table 3**). Cases were: 2 clear cell sarcoma, 1 fibrosarcoma, 1 malignant tumor of nerve sheath and 1 metastatic adenocarcinoma.

**Table 3:** Ct values of qPCR amplified results

**Case no.**	Sample*	**Reference gene Ct **	**Sample Ct**
11	N	9.08	-
	T	4.67	40.64
12	N	5.44	-
	T	3.87	45.07
13	N	12.55	-
	T	4.52	39.56
14	N	5.43	-
	T	4.21	48.05
22	N	8.09	-
	T	7.49	41.17
* N = normal tissue RNA, T = tumor RNA			

In order to distinguish non-specific amplification, the five cases were analyzed in terms of technical conditions and tumor characteristics (**Table 4**). The technical conditions of the test were appropriate and the appearance of the amplification curve was similar to that of the reference genes. In addition, considering that the tumors showed high malignant potential, we concluded that these amplifications could be interpreted to represent rather weak positive results than non-specific amplifications.

**Table 4:** Characteristics of 5 cases with qPCR weak amplification

**Case no.**	**Final diagnosis**	**Age**	**Gender**	**Periosteal or bone shaft invasion**	**Metastasis**
11	Fibrosarcoma	70	M	No	No
12	Clear cell sarcoma	31	F	No	No
13	Clear cell sarcoma	58	F	No	No
14	Metastatic adenocarcinoma	47	M	Yes (periosteum, shaft)	-
22	Malignant tumor of nerve sheath	56	F	No	No

## Discussion

Although characteristic to ES, the EWS/FLI-1 fusion may also occur with a lower frequency in other types of tumors (clear cell sarcoma, PNET, Askin tumor of the chest wall). Therefore, it is interesting to study the occurrence of the fusion in clinically diagnosed sarcomas belonging to other categories of connective tissue tumors.

The EWS/FLI-1 fusion presence is achieved by molecular methodology with qPCR [**17**], by using the RNA extracted from the tumor material as a biological sample. The identification of the gene modification is made by means of a primer consisting of a specific short chain of nucleotides complementary to a target sequence on the fused gene [**18**]. If the fusion is present, the probe is engaged in the target sequence, whose copies are amplified.

According to our results, 5 cases of 20, assessed by qPCR for EWS/FLI-1 fusion, showed gene amplifications occurring in the marginal range (“gray area”) of positivity (Ct between 39-45). To differentiate between unspecific amplification and a weak positive result, each sample was analyzed in terms of technical standpoint (test parameters), the patient's clinical course and the tumor appearance on physical and intraoperative examination. The histopathological examination was considered as a corollary of these data, confirming the correctness of clinical assumptions and molecular biology data.

The analysis of histopathological diagnoses of the five cases revealed that the patients had different types of soft tissue tumors, but not ES (**Table 3**). In 4 of these cases, the presence of a tumor from Ewing family or the other above cited soft tissue tumors was found. These types of tumors are mentioned in literature and may present EWS/FLI-1 fusion [**12**,**14**-**16**]. 4 primary tumors showed no signs of invasion in the periosteum or bone shaft and no metastases, while 1 tumor was metastasis itself.

Several conditions would explain why the PCR amplification started later, at the limit of the kit cycles range. Considering the clinical and histopathological data, it may be concluded that the tumors were in a relatively early or medium stage of development. Consequently, the fusion may not be represented in all tumor cells, but only in a small number of them with increased malignant potential due to this fusion. It is possible that the number of tumor cells was not sufficient to generate a massive presence of mutant RNA sequences. Furthermore, if the tumors were not ES, the degree of fusion could be reduced, these tumors being characterized by a significant presence of other types of translocations not investigated in this study: t(9,22), t(11,22), t(12,22), t(12,16), t(X,18) [**19**].

One positive case was a secondary tumor, metastasis of an adenocarcinoma with an undetermined location. Thus, there was an interesting problem, namely whether the fusion could occur in the epithelial tumors with metastases in the soft tissue. We suggested that the fusion could occur in these tumors as a result of local factors in which metastasis developed (subcutaneous tissue, fat, bone, cartilage). It could be an example of transformation under the influence of tumor environment.

In the cases investigated, the diagnosis of sarcoma was first clinically established, but it was difficult to make a distinction regarding the variety of sarcoma. This diagnostic completion is generally provided by histopathological examination. If the tumor is excised, or even before surgery (when biopsy is practiced), genomic investigation may establish the malignancy of the tumor and its possible affiliation earlier than histopathology. It can determine whether the clinically diagnosed tumor is part of the group that presented the EWS/FLI-1 fusion.

This paper suggests that the genomic investigation of patients with soft tissue tumors is useful to guide the diagnosis before the histopathological examination. It should be considered that histopathology can sometimes be ambiguous in terms of proliferating cell type, and the tumor may fall into several categories of sarcomas. As a result, these considerations can justify the proposal of the screening of EWS/FLI-1 fusion in a larger variety of soft tissue tumors. In this case, besides the immunohistochemical assays, genomics investigation may determine tumor malignancy.

**Acknowledgement**

This paper is partly supported by the Sectorial Operational Programme Human Resources Development (SOPHRD), financed by the European Social Fund and the Romanian Government under the contract number POSDRU 141531 (a fellowship awarded to Radu Huică, MD).

**Conflict of interest**

The authors declare that they have no conflict of interest.
